# The use of ball pits and playpens in laboratory Lister Hooded male rats induces ultrasonic vocalisations indicating a more positive affective state and can reduce the welfare impacts of aversive procedures

**DOI:** 10.1177/00236772211065920

**Published:** 2022-01-13

**Authors:** Justyna K Hinchcliffe, Megan G Jackson, Emma SJ Robinson

**Affiliations:** School of Physiology, Pharmacology and Neuroscience, 1980 University of Bristol, UK

**Keywords:** Rodents, organisms and models, laboratory animal welfare, ethics and welfare, refinement, environmental enrichment, vocalization, behaviour

## Abstract

The advancement and quality of science rely on research that is robust and unbiased in its experimental design, execution, analysis, and reproducibility. In preclinical research, a better understanding of animal emotions and refinement of their husbandry, housing, and handling are important goals in providing good animal welfare in a laboratory setting which underpins rigorous research quality. Induction of positive emotional state in animals is a key component of their well-being, and one approach is to increase their environmental complexity using, for example, ball pits or playpens in rats. In this study, we recorded 50 kHz ultrasonic vocalisations (USVs) during animals’ exposure to the ball pit and playpen. We have previously shown that 50 kHz USVs provide a graded and quantifiable measure of an animal’s emotional state, and here find that access to the ball pit and playpen increases 50 kHz USVs, indicative of a more positive affective state. Using our affective bias test (ABT) we next quantified the animals’ emotional response to an aversive intervention and whether this could be attenuated by access to a playpen. The playpen exposure completely mitigated the negative affective state induced by an anxiogenic drug when compared with animals who experienced the drug in the home cage. Together, these findings suggest ball pits and playpens provide a simple and effective method to improve the welfare of laboratory rats and reduce the cumulative suffering they experience from their housing conditions and minor, aversive procedures.

## L’utilisation de fosse à balles et de zones de jeu dans les laboratoires contenant des rats Lister Hooded induit des vocalisations ultrasoniques qui indiquent un état affectif positif et peuvent réduire l’impact des procédures aversives sur le bien-être des animaux.Résumé

L’avancement et la qualité de la science dépendent d’une recherche robuste et impartiale au niveau de sa conception, de son déroulement, de son analyse et de sa reproductibilité. En recherche préclinique, une meilleure compréhension des émotions animales et un raffinement de l’élevage, du logement et de la manipulation des animaux sont des objectifs importants pour aboutir à un meilleur bien-être des animaux de laboratoire, afin d’appuyer une recherche de qualité. L’induction d’un état émotionnel positif chez les animaux s’avère donc une composante clé du bien-être. L’une des approches à adopter à cet effet consiste à augmenter la complexité de leur environnement en utilisant, par exemple, des fosses à balles ou des zones de jeu pour les rats. Dans cette étude, nous avons enregistré des vocalisations ultrasoniques (VUS) de 50kHz lorsque les animaux étaient dans la fosse à balles ou dans la zone de jeu. Nous avons précédemment montré que des VUS de 50kHz fournissaient une mesure graduée et quantifiable de l’état émotionnel d’un animal. L’accès à la fosse à balles et à la zone de jeu augmente le nombre de VUS de 50kHz, ce qui indique un état affectif plus positif. En utilisant notre test du biais affectif (TBA), nous avons ensuite quantifié la réponse émotionnelle des animaux à une intervention aversive et cherché à savoir si elle pouvait être atténuée par un accès à la zone de jeu. Par rapport aux animaux ayant fait l’expérience du même médicament, mais en cage, l’exposition à la zone de jeu a complètement atténué l’état affectif négatif induit par un anxiogénique. Ensemble, ces résultats suggèrent que les fosses à balles et les zones de jeu fournissent une méthode simple et efficace pour améliorer le bien-être des rats de laboratoire et réduire la souffrance cumulative qu’ils éprouvent en fonction de leurs conditions de logement et des procédures mineures ou aversives.

## Der Einsatz von Kugelbädern und Laufställen bei männlichen Lister-Hooded-Laborratten führt zu Ultraschallvokalisationen, die einen positiveren affektiven Zustand anzeigen und die Auswirkungen aversiver Verfahren auf das Wohlergehen der Tiere verringern können. Abstract

Fortschritt und Qualität der Wissenschaft bedingen eine Forschung mit robuster und objektiver Versuchsplanung, -durchführung, -analyse und -reproduzierbarkeit ab. In der präklinischen Forschung sind ein besseres Verständnis der Emotionen von Tieren und die Verbesserung ihrer Haltung, Unterbringung und Handhabung wichtige Ziele, um guten Tierschutz in einer Laborumgebung zu gewährleisten, welches eine stringente Qualität der Forschung bedingt. Die Auslösung positiver emotionaler Zustände bei den Tieren ist ein Schlüsselelement für ihr Wohlbefinden, und ein diesbezüglicher Ansatz besteht in der Erhöhung der Komplexität ihrer Umgebung, z. B. mit Hilfe von Kugelbädern oder Laufställen für Ratten. In dieser Studie haben wir 50-kHz-Ultraschallvokalisationen (USVs) aufgezeichnet, während die Tiere Kugelbad und Laufstall ausgesetzt waren. Wir haben bereits früher gezeigt, dass 50-kHz-USVs ein differenziertes und quantifizierbares Maß für den emotionalen Zustand eines Tieres darstellen, und haben hier festgestellt, dass der Zugang zu Kugelbad und Laufstall die 50-kHz-USVs erhöht, was auf einen positiveren affektiven Zustand hinweist. Mit unserem Test zur affektiven Verzerrung (ABT) haben wir sodann die emotionale Reaktion der Tiere auf eine aversive Intervention quantifiziert und versucht festzustellen, ob diese durch Zugang zu einem Laufstall abgeschwächt werden kann. Der Laufstall milderte den negativen affektiven Zustand, der durch eine angstauslösende Droge herbeigeführt wurde, vollständig ab, verglichen mit Tieren, die die Droge im Heimkäfig erhielten. Diese Ergebnisse legen nahe, dass Kugelbäder und Laufställe eine einfache und wirksame Methode sind, um das Wohlergehen von Laborratten zu verbessern und kumuliertes Leiden zu verringern, dem sie aufgrund ihrer Haltungsbedingungen und kleinerer, aversiver Verfahren ausgesetzt sind.

## El uso de piscinas de bolas y corralitos de juego con ratas macho Lister Hooded de laboratorio induce a vocalizaciones ultrasónicas lo que indica un estado afectivo más positivo que podría reducir los impactos en el bienestar del uso de procedimientos aversivos. Resumen

El avance y la calidad de la ciencia dependen de que la investigación sea sólida e imparcial en su diseño experimental, ejecución, análisis y reproducibilidad. En la investigación preclínica, una mejor comprensión de las emociones de los animales y el perfeccionamiento de su cría, enjaulamiento y manipulación son objetivos importantes para proporcionar un buen bienestar a los animales en un entorno de laboratorio que sustente una investigación rigurosa de calidad. La inducción de un estado emocional positivo en los animales es un componente clave de su bienestar y uno de los métodos consiste en aumentar la complejidad de su entorno utilizando, por ejemplo, piscinas de bolas o corralitos de juego para los roedores. En este estudio, registramos unas vocalizaciones ultrasónicas de 50 kHz (USV) durante la exposición de los animales a la piscina de bolas y al corralito. Anteriormente hemos demostrado que las USV de 50 kHz proporcionan una medida graduada y cuantificable del estado emocional de un animal y aquí encontramos que el acceso a la piscina de bolas y al corralito aumenta las USV de 50 kHz, lo que indica un estado afectivo más positivo. A continuación, mediante nuestra prueba de predisposición afectiva (ABT), medimos la respuesta emocional de los animales a una intervención aversiva y si esta podía atenuarse mediante el acceso a un corralito. La exposición al corralito mitigó completamente el estado afectivo negativo inducido por un fármaco ansiógeno en comparación con los animales que recibieron el fármaco en su jaula doméstica. En conjunto, estos estudios sugieren que las piscinas de bolas y los corralitos de juego proporcionan un método sencillo y eficaz para mejorar el bienestar de los roedores de laboratorio y reducir el sufrimiento acumulado que experimentan por sus condiciones de enjaulamiento y los procedimientos menores y aversivos.

## Introduction

Emotional states in non-human animals comprise multiple physiological, behavioural, and cognitive components.^1,2^ The ability to accurately and reliably quantify the affective state of laboratory rodents is critical to understanding animal welfare. The induction and measurement of positive animal emotions are key to the improvement of their welfare, methods of housing, handling, and general husbandry. Rats emit ultrasonic 50 kHz ultrasonic vocalisations (USVs) during or in anticipation of juvenile play, mating, food consumption, and during human-simulated play or ‘tickling’.^3,4^ These high-frequency vocalisations are assumed to reflect the animals’ positive emotional state and are a good indicator of animal welfare.^5,6^

To objectively test the relationship between positive affect and 50 kHz calls, in our previous study we combined a well-validated and translational assay, the affective bias test (ABT) readout, with individual animals’ USV calls during tickling.^7^ Rats that emitted the most calls had the highest positive emotional response to tickling, but those who emitted no or few calls did not show a positive response, and there was a direct correlation between calls and positive affect.^7^ Integrating the behavioural readout from the ABT and tickling-induced 50 kHz calls suggested that USVs alone can provide researchers with a simple, graded, and quantifiable measure of their individual affective experience.

New welfare guidelines by the National Centre for the Replacement, Refinement and Reduction of Animals in Research (NC3Rs) encouraged the researchers and animal technicians to tickle rats as a refined additional method for animal handling.^8^ Recently a new form of enrichment for laboratory rats, the use of playpens, has been suggested as an additional method of refinement.^9^ It has been suggested that frequent use of playpens can be beneficial for laboratory rats and contribute to a more positive affective state and better welfare. Although this approach shows qualitative benefits, objective scientific evidence has yet to be established. Playpens are large enclosures with a complex, enriched environment that provides laboratory rats with opportunities to socialise and exercise, and promotes naturalistic behaviours such as digging, rearing, and climbing. The idea is to give routine access to increased vertical and horizontal space that standard rat cages are not able to provide. The study by Makowska and Weary^10^ demonstrated evidence that space restrictions of standard rat housing may lead to the poor physical and psychological health of the animals. The ability to express natural behaviours, exercise, and rat socialisation is critical to their quality of laboratory life and well-being. Authors measured that in semi-naturalistic caging (91 × 64 × 125 cm) young rats burrowed approximately 30 times per day, climbed 75 times per day, and stood upright 180 times per day. Rats in standard caging (45 × 24 × 20 cm) were engaged in more lateral stretching to compensate positional stress associated with restricted movement. The idea and implementation of playpens in a laboratory environment are not novel, as they are widely used in non-human primate, dog, and rabbit research facilities.^11–15^ The concept is similar: frequent access to environmentally enriched playpens to give animals a stimulating positive experience, to minimise their stress levels, to enable them to express more natural behaviours, and to provide daily activities to reduce boredom. The use of large, complex enclosures reduced the number of stereotypies in rabbits^11,12^ and dogs,^13^ and reduced aggression in primates.^14,15^

The aim of this study was to investigate the affective experience of rats exposed to two types of playpens using their emission of USVs as a representation of their affective state. To further explore the potential benefits of playpens we tested if the access to playpen could mitigate FG7142-induced negative bias in rats. FG7142 (benzodiazepine partial inverse agonist) is a validated anxiogenic and pro-depressant compound that reliably induces a negative affective bias in rats.^16^ To test this, we used the ABT, which has demonstrated the predicted affective valence for a wide range of pharmacological, immune, hormonal, and environmental manipulations, generating medium to large effect sizes.^16–18^

## Methods

### Animals and housing

Male Lister Hooded rats (Harlan, UK) (*N* = 46) from four cohorts were used in these experiments. No information was available from the supplier about the litter status of the animals and so it is not known whether any of the animals were litter mates. All rats were weighing approximately 300–400 g (age: 13–16 weeks) during the USVs recording sessions. For the ABT six rats from cohort 3 were used and at the beginning of the training their weights were ∼300–350 g. In keeping with the requirements to minimise the numbers of animals needed specifically for the USVs recording study, rats that had been brought in for other planned studies were utilised. Without adversely affecting our planned experiments, all animals were tested in the same period of an experimental break and were not undergoing any procedures or treatments. Cohort 1 (*N* = 16) was used for operant conditioning work, cohorts 2 (*N* = 12) and 3 (*N* = 14) were used for a learning and memory task, and cohort 4 (*N* = 4) was used for surgical manipulations. All animals were pair-housed in environmentally enriched laboratory cages (55 × 35 × 21 cm) with aspen woodchip bedding, paper bedding, cotton rope, wood block, cardboard tube (Ø 8 cm) and red Perspex® house (30 × 17 × 10 cm), under a 12:12 h reverse light–dark cycle (lights off at 08:00 h) and in temperature-controlled conditions (21 ± 1°C). Rats were food restricted to approximately 90% of their free feeding weights matched to the normal growth curve (∼18 g of laboratory chow (Purina, UK) per rat was placed in their cage food hopper and all rats were fed once at the end of the experimental day) and provided with *ad libitum* water. The behavioural procedures and testing were performed during the animals’ active phase between 09:00 h and 17:00 h. All animals were given daily health and welfare checks by the animal facility technicians and the researchers. All experimental procedures were conducted in accordance with the UK Animals (Scientific Procedures) Act 1986 and were approved by the University of Bristol Animal Welfare and Ethical Review Body and UK Home Office (PPL number P9B6A09A1).

### Recording of USVs

#### Recording equipment

USVs were recorded during four sessions in the ball pit or playpen and two sessions in the control arena by using a high-frequency microphone (2–200 kHz range, CM16/CMPA UltraSoundGate Condenser Microphone, Avisoft Bioacoustics, Germany) suspended 50 cm above the floor of the experimental arena and connected to a computer via an ultrasound recording interface (116H UltraSoundGate, Avisoft Bioacoustics, Germany), and acoustic data were displayed in real time by Avisoft RECORDER USGH (version 4.2., Avisoft Bioacoustics, Germany). To evaluate USV data, spectrograms were generated in Deep Squeak (version 2.6.2.) and randomly cross checked in the SASLab Pro (version 5.2., Avisoft Bioacoustics, Germany) to confirm accuracy of the analysis. The criteria for call identification were set at frequencies between 35 and 90 kHz and duration 10–150 ms for flat and frequency-modulated 50 kHz calls, and frequencies between 18 and 32 kHz and duration 300–3000 ms for 22 kHz calls. To reduce background noise outside the relevant frequency band low- (below 18 kHz) and high-pass (above 90 kHz) filters were used. The numbers of 50 kHz calls and 22 kHz calls were batch analysed in Deep Squeak by using a ‘supervised classification’ established by an experienced experimenter (JKH). All USVs recorded were found to be in the 50 kHz range with no 22 kHz calls detected. USVs data collected during the ball pit, playpen, and control arena sessions were analysed with the researcher blinded to the animals’ batch ID.

#### Ball pit and playpen arena

**Figure 1.**
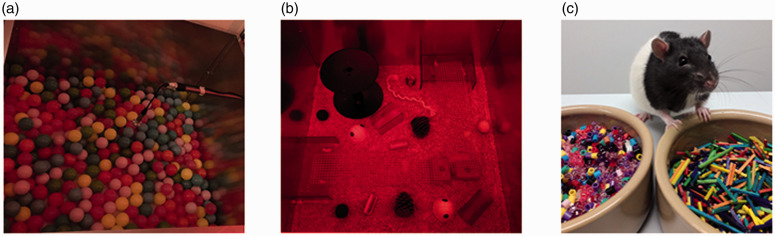
Photograph of ball pit (panel a), playpen (panel b) and the affective bias test (panel c). Panel (a) shows a ball pit arena (100 × 100 × 60 cm) with 400 plastic balls and panel (b) shows a playpen arena (100 × 100 × 60 cm) with a wide range of toys and enrichment. Panel (c) illustrates the affective bias test set-up (photograph taken in normal lighting for illustrative purpose only). The assay involves training rats to associate finding a food reward in a specific digging substrate. Each of the two experiences is learnt independently and with a manipulation of affective state before one of the sessions. Bias is then measured using a preference test where rats are ‘asked’ which substrate they associate with a better outcome (for experimental details see, Stuart et al.^16^ and Hinchcliffe et al.^18^).

The testing was carried out in a metal arena with white Perspex® floor (100 × 100×60 cm), which was converted either into a ball pit or playpen. The ball pit consisted of 400 plastic balls (see Figure 1(a)). The floor of the playpen was covered with aspen woodchip bedding, and the arena was furnished with various toys: a circular polyvinyl platform (Ø 28 cm and height 25 cm), two metal ladders (60 × 13 cm), two autoclaved fern cones (Ø 9 cm and height 10 cm), two rectangular Perspex® platforms (30 × 17 × 10 cm), two tennis balls made of rubber and covered with green felt (Ø 6 cm), two blue polyvinyl tumble dryer balls (Ø 6 cm), two wooden scrubbing brushes (9 × 4 cm), one red Perspex® tube (Ø 8 cm and length 15 cm), one red Perspex® square tunnel (Ø 8 cm and length 15 cm), two white cardboard houses (Ø 10 cm), two red polycarbonate houses (Ø 10 cm), two white cotton ropes (15 cm) (see Figure 1(b)). Care was taken to select objects which would not cause harm to the animals even if they chewed or ingested any of the items. For the control recordings an empty clean arena was used to provide USV data for the arena context but in the absence of enrichment.

#### Recording of USVs in control, ball pit, and playpen

In this study block randomisation was used; for each batch half of the animals were randomly assigned to the ball pit group and the other half to the playpen group. The sub-blocks (ball pit or playpen) of animals for each batch were then added together as one group for ball pit (*N* = 11) and the other group for playpen (*N* = 12). For all recording sessions, rats were transported in pairs (cage mates) to the behavioural room in a white transport bucket (44 × 23 × 19 cm) filled with woodchip bedding. The behavioural room was furnished with a ball pit/playpen/control arena on one side, and a storage unit and a table with a computer recording USVs on the other side of the room. The computer was ∼2 m away from the testing arena, and sound was attenuated with polystyrene sheets to reduce noise and USVs that can be potentially emitted by the electronic parts, such as the computer screen. All animals were tested under red lighting conditions and mechanical ventilation system with air exchange rate at 23–28 per hour. Each recording consisted of 5 min in the control arena and ball pit or control arena and playpen. All four sessions took place on separate days. USVs were recorded from two rats (cage mates) to minimise the number of 50 kHz flat calls (social-coordinating or contact calls) and focus on the 50 kHz frequency-modulated calls associated with reward and positive affective state. During playpen recordings woodchip was scattered on the arena’s floor from each batch and kept consistent to avoid additional olfactory factors disrupting the emission of USVs. During control sessions the floor of the arena was cleaned between each rat pair to avoid any confounding olfactory cues. Following each recording, rats were returned to their home cage.

### ABT

The ABT testing was carried out in a Perspex® arena (40 × 40 cm) with two ceramic bowls (Ø 10 cm) and a trio of digging substrates (reward-paired substrates – ‘A’ or ‘B’ versus unrewarded substrate – ‘C’, matched for digging effort and counterbalanced across subjects, see Figure 1(c)). The training and testing for the ABT were similar to those previously described in Hinchcliffe et al.^7,18^ Testing consisted of four *pairing sessions* (one per day) followed by a *choice test* on the fifth day of the same week. During pairing sessions, each trial involved presenting the rat with a choice between two bowls containing two different digging substrates, one of which was reward-paired (substrate ‘A’ or ‘B’) and contained a single 45 mg food pellet, and the other of which was unrewarded (substrate ‘C’). Substrate ‘C’ was kept the same for all four pairing sessions and a pellet was crushed into the bowl and mixed within the substrate, to prevent choices based on odour. One of substrates A or B was presented during pairing sessions on days 1 and 3, and the other was presented on days 2 and 4, with order counterbalanced across subjects and manipulations. The number of trials to reach criterion (six consecutive correct choices for the reward-paired substrate) and latency to dig within each pairing session, and latency to dig during choice test are recorded for each animal.

#### The effects of playpen on FG7142-induced negative affective bias

**Figure 2.**
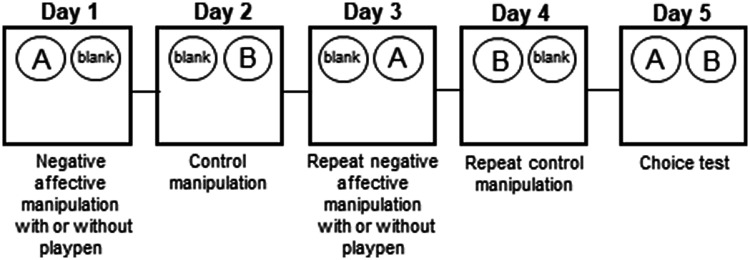
Overview of affective bias test study design for experiment 2. Figure illustrates protocol to test acute treatment effects on new learning and development of the affective state-induced biases. Animals undergo pairing sessions with drug treatment with/without manipulation (e.g. on days 1 and 3) versus control treatment (e.g. on days 2 and 4). The development of the affective biases is then measured by the choice test (day 5).

This experiment was composed of a 2-weeks counterbalanced within-study design where rats underwent both manipulations. One manipulation (e.g. on day 1 and 3) was a treatment with FG7142 (3 mg/kg, SC) 30 min prior to reward-substrate ‘A’ (or ‘B’) versus ‘C’ pairing session (see Figure 2). The other manipulation was also FG7142 treatment prior to reward-substrate pairing, but it was followed by 1 h in the playpen with the cage mate. The animals were placed in the playpen around ∼3 h after the pairing session. The effects of playpen on FG7142-induced negative affective bias were quantified during the choice test on day 5 when the two previously rewarded substrates (‘A’ and ‘B’) were presented at the same time for 30 trials. In order to keep rats motivated to continue choosing without providing new associative information, a single food pellet was placed in either bowl using a random schedule with a probability of one in three so that rats randomly received a reward. Both bowls also had a pellet crushed and placed in the substrate to reduce the likelihood of the animal using odour to find the reward. The experimenter was blind to drug treatment.

### Data analysis

Data were analysed and figures were created using GraphPad Prism 8.4 (GraphPad Software, USA). Each individual data point indicates vocalisations number from a pair of rats placed in the arena. USVs between the manipulation groups were analysed using repeated measures ANOVA for control versus ball pit analysis and mixed-effects model ANOVA for control versus playpen analysis. USVs could not be recorded from three rat pairs (*N* = 3) in the control sessions (playpen analysis) due to surgical manipulations they had undergone at that time. Repeated measures ANOVA cannot handle missing values, therefore the data were analysed by fitting a mixed model as implemented in GraphPad Prism 8.4. In the absence of missing values, this method gives the same *p*-values and multiple comparisons tests as repeated measures ANOVA. Post hoc analysis utilised a Dunnett’s multiple comparison test.

Choice bias score was calculated as the number of choices made for the drug-paired substrate divided by the total number of trials multiplied by 100 to give a percentage value. A value of 50 was then subtracted to give a score where a choice bias towards the drug-paired substrate gave a positive value and a bias towards the control-paired substrate gave a negative value. Choice bias scores and response latency during the choice test were analysed utilising a paired *t*-test between ‘home cage’ or ‘playpen’ group. Positive or negative affective biases were also analysed using a one-sample *t*-test against a null hypothesised mean of 0% choice bias. For each animal, mean latency to dig and trials to criterion during ABT pairing sessions were analysed using a paired *t*-test. The generated datasets can be available with this article online.

## Results

### Experiment 1: The effects of ball pit and playpen on the emission of 50 kHz USVs in male rats

**Figure 3.**
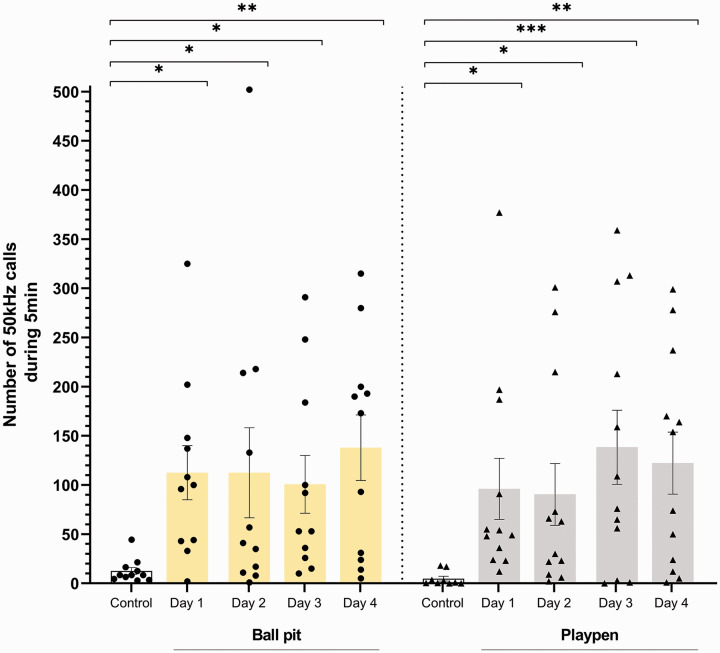
The effects of ball pit and playpen on the emission of 50 kHz ultrasonic vocalisations in Lister Hooded male rats. In both conditions, ball pit (day 1: *p* = 0.0215 vs. control, day 2: *p* = 0.0216 vs. control, day 3: *p* = 0.0492 vs. control, day 4: *p* = 0.0030 vs. control) and playpen (day 1: *p* = 0.0244 vs. control, day 2: *p* = 0.0375 vs. control, day 3: *p* = 0.0006 vs. control, day 4: *p* = 0.0026 vs. control), rats vocalised with 50 kHz calls significantly more than in the control (empty arena) condition. There were no changes observed in number of USVs over time, suggesting the animals positive affective experience did not diminish with repeated exposures. Data shown as average number ± SEM of 50 kHz calls over 2 days (control: illustrated by white bar) and number of 50 kHz calls emitted over individual days (ball pit: illustrated by yellow bars or playpen: illustrated by grey bars) and individual data points (ball pit group: illustrated by circle symbols or playpen group: illustrated by triangle symbols), *N* = 9–12 per group, *p < 0.05, **p < 0.01, ***p < 0.001 (Dunnett’s test).

A main effect of manipulation, i.e. access to the ball pit (*N* = 11, RM ANOVA, F4.40 = 3.886, *p* = 0.0093) or access to playpen (*N* = 9–12, mixed-effects model ANOVA, F4.41 = 4.936, *p* = 0.0024), was found on number of 50 kHz USVs emitted by rats, compared with the control session (Figure 3). Post hoc analysis using Dunnett’s multiple comparison test showed that animals vocalised with 50 kHz calls significantly more during the ball pit sessions in all four days (day 1: *p* = 0.0215 vs. control, day 2: *p* = 0.0216 vs. control, day 3: *p* = 0.0492 vs. control, day 4: *p* = 0.0030 vs. control). Similar findings were observed during playpen sessions: rats emitted a greater number of 50 kHz USVS during four sessions (day 1: *p* = 0.0244 vs. control, day 2: *p* = 0.0375 vs. control, day 3: *p* = 0.0006 vs. control, day 4: *p* = 0.0026 vs. control). No 22 kHz alarm calls were recorded. When detailed analysis of USVs was performed, the majority of calls recorded in ball pit and playpen were represented by frequency-modulated 50 kHz (see Table 1), indicating a positive affective state in rats.

**Table 1.** Percentage of flat and frequency-modulated (FM) calls out of all 50 kHz USVs recorded during 5 min sessions.  Only rat pairs that emitted 50 kHz calls were included in this analysis (^a^day 1–2: *N* = 4–7; ^b^day 1–4: *N* = 11; ^c^day 1–2: *N* = 3–4; ^d^day 1–4: *N* = 9–12).

	Manipulations							
	Control^a^		Ball pit^b^		Control^c^		Playpen^d^	
	Flat50 kHz calls	FM50 kHz calls	Flat50 kHz calls	FM50 kHz calls	Flat50 kHz calls	FM50 kHz calls	Flat50 kHz calls	FM50 kHz calls
Day 1	3.1	96.9	5.70	94.30	25.0	75.0	3.23	96.77
Day 2	5.8	94.2	3.08	96.92	0.0	100.0	4.68	95.32
Day 3	n/a	n/a	3.10	96.90	n/a	n/a	2.13	97.87
Day 4	n/a	n/a	2.63	97.37	n/a	n/a	5.71	94.29

### Experiment 2: The effects of playpen on FG7142-induced negative bias in male rats

**Figure 4.**
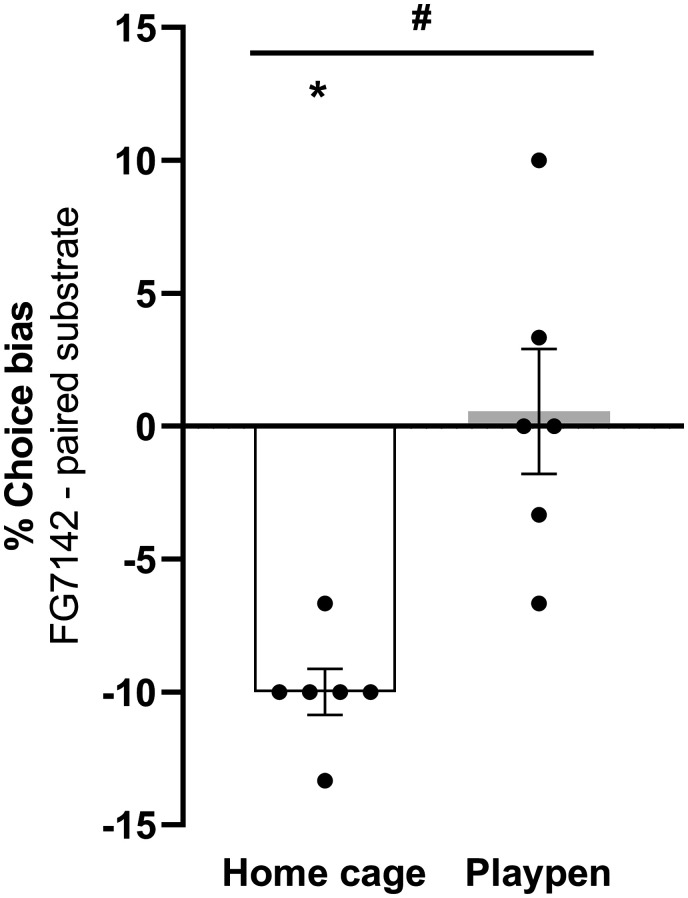
The effects of playpen on FG7142-induced negative bias in Lister Hooded male rats. Consistent with previous studies, FG7142 administration induced a negative affective bias in rats in the home cage group but this effect was attenuated in rats which experienced the playpen for 1 h after the drug treatment and substrate-reward pairing session. Data shown as mean ± SEM and individual data points, *N* = 6 per group, *p < 0.05 (one-sample *t*-test versus theoretical mean of 0% choice bias), ^#^*p* < 0.05 (post hoc paired *t*-test).

Following treatment with FG7142, rats in the home cage group developed a significant negative affective bias (one-sample *t*-test: t_5_ = 11.62, *p* < 0.0001, Figure 4). In the other group, rats kept in the playpen for 1 h following reward-substrate pairing session showed a significant attenuation of the negative bias (paired *t*-test: t_5_ = 3.630, *p* = 0.0151, Figure 4). There were no significant changes during pairing sessions, either on response latency to dig or number of trials to criterion between the groups; also no difference in latency to make a choice during the ABT choice test was found, indicating that non-specific effects, i.e. changes in motivation or sedation, were not observed (see Table 2).

**Table 2.** Experiment 2: number of trials to criterion and latency to dig during pairing sessions, and latency to make choice in the ABT choice test. Data shown as mean (*N* = 6 animals/group) ± SEM averaged from the two pairing sessions for each substrate-reward association and from choice test.

Manipulation		Response latency (s)		Trials to criterion	
PAIRING SESSIONS		vehicle	FG7142	vehicle	FG7142
FG7142 + home cage/playpen	Week 1	1.8 ± 0.2	2.1 ± 0.2	6.2 ± 0.1	6.5 ± 0.2
	Week 2	1.7 ± 0.1	1.8 ± 0.1	6.1 ± 0.1	6.3 ± 0.2
Manipulation					
CHOICE TEST		Response latency (s)			
Home cage		1.6 ± 0.1			
Playpen		1.5 ± 0.0			

## Discussion

Our study demonstrated that animals emitted a higher number of frequency-modulated 50 kHz calls during sessions in the ball pit and playpen compared with the control conditions, indicating that time in the ball pit or playpen induces a more positive affective state. Utilising recordings of 50 kHz USVs as a graded and quantifiable measure of an animal’s affective state, we showed that access to the ball pit and playpen is a positive experience for all animals as no alarm calls were identified. In the second study, animals which had access to the play pen after receiving an aversive drug treatment showed an attenuated negative affective state when measured in the ABT, suggesting that the playpen could reduce the negative impacts of this treatment. These data provide quantitative evidence supporting the welfare benefits of ball pits and playpens for laboratory rats.

The large number of frequency-modulated 50 kHz calls emitted by rats during either the playpen or ball pit experience indicates that the animals experience this with a positive affective valence. We have previously shown that a similar social and environmental enrichment procedure is associated with a positive affective bias in the ABT,^16,18^ and others have reported that use of complex and enriched housing decreases aggressive behaviours,^19^ induces neural development,^20^ increases dendrite complexity,^21,22^ reduces heart rate and corticosterone levels,^23^ and is associated with improvements in overall health and well-being^24,25^ in rats. Interestingly, animals did not emit 22 kHz calls in any of the arenas, suggesting none of these novel environments was aversive or anxiogenic. The lack of 22 kHz alarm calls is most likely explained by the habituation to the arena prior to testing, and when used as the neutral context it was not aversive in rats. It would also appear that complexity of the environment is important, as the ability to exercise in the empty arena was not enough to generate a positive experience. The social interaction along with the exploration of an enriched playpen or ball pit would seem to be crucial to generation of the positive emotional state in rats.

Treatments linked to negative mood in humans, e.g. FG7142, or the stress hormone cortisol (corticosterone in rats), as well as psychosocial stress reliably induce a negative affective bias in the ABT in male and female rats.^16–18^ In this study, negative affective state induced by FG7142 was attenuated by the post-treatment exposure to the playpen. Interestingly, we have also seen a similar attenuation of a negative affective bias following treatment with the rapid-acting antidepressant ketamine when it was administered immediately before the choice test.^17^ Although further studies utilising a larger sample size are needed to test the reproducibility of our findings, the results presented here suggest that even a short period of time in a ball pit or playpen could be used to reduce the negative consequences of aversive procedures in rats. Our study only looked at a 5 min exposure, and studies using longer periods of time and repeated exposure would be of interest and may provide greater benefits. This may be particularly useful for reducing the cumulative suffering of animals experiencing repeated procedures such as chronic dosing in toxicology studies and where the specific experimental objectives are not dependent on the arising stress or affective state changes these procedures induce.

Our data have shown that rodents find the ball pit and playpen a positive affective experience, and this has a number of implications within the wider academic, scientific, and industrial settings. Enabling rats to express more naturalistic behaviours and regular opportunities for a positive affective experience may reduce the development of some of the commonly observed negative behaviours which develop in laboratory mice such as stereotypies. The ball pit or playpen also provide an environment where the animals can express natural behaviours, such as stretching, rearing, and climbing, which are commonly restricted in standard caging. This is especially the case for fully grown adult animals where limited floor space and cage height can restrict these behaviours. Ball pits and playpens can also be beneficial for rats undergoing procedures that require individual housing (in studies involving telemetry, cannulations, or implants), as they can enable periods of social interactions under human supervision without triggering aggressive and stressful encounters. Frequent access to ball pits or playpens is beneficial not only for laboratory rats but also for researchers and animal technicians. Animals are handled more often and therefore become easier and less anxious to handle, experience more positive and rewarding interaction with the handler, and demonstrate reduced aversion towards the handler.^9^ There are, however, several scientific considerations and potential disadvantages that may discourage or limit the implementation of playpens in the wider research community. One issue is the financial costs and space in the animal facility, which may limit researchers’ abilities to set-up long-term playpens. There is also the potential for challenges to biosecurity and, whilst the playpen and objects can be designed to be easily cleaned, this takes time, and the setting up of multiple playpens, i.e. for each holding room, is unlikely to be feasible. Another important factor to consider is the lack of published data on the beneficial effects of ball pits or playpens on animal well-being versus the experimental study endpoints. Ball pits and playpens are not advised to be used for some disease animal models, e.g. animal models of depression or chronic pain, due to obvious confounds in terms of their effects on animals’ emotional state.

Refined handling techniques and habituation to the researchers or animal technicians, and housing conditions,^26,27^ including the use of ball pits and playpens, offer the potential to reduce the mild but cumulative negative affective experiences of rats housed in laboratory conditions, and these results provide objective data supporting the welfare benefits of ball pits and playpens. Animals living in an enriched environment with lower stress levels and improved well-being should provide a better model for research, and are likely to generate more reliable and reproducible data with less variability.^11,26^
